# Association of handgrip strength with health care utilisation among older adults: A longitudinal study in China

**DOI:** 10.7189/jogh.14.04160

**Published:** 2024-08-30

**Authors:** Yueyue You, Xiaobing Wu, Ziyang Zhang, Fengzhu Xie, Yali Lin, Deliang Lv, Zhiguang Zhao

**Affiliations:** Cardio-Cerebrovascular and Diabetes Prevention and Control Department, Shenzhen Centre for Chronic Disease Control, Shenzhen, China

## Abstract

**Background:**

Evidence on the association between handgrip strength (HGS) and health care utilisation among Chinese older adults is scarce. In this study, we aimed to investigate the association of HGS with health care utilisation and to identify whether these associations varied by gender.

**Methods:**

The analytic sample of this prospective cohort study included 6007 Chinese older adults (≥60 years) from 2011 to 2018 waves of the China Health and Retirement Longitudinal Study. A handgrip dynamometer was used to measure HGS. We measured health care utilisation by outpatient visits, inpatient visits, and unmet hospitalisation needs. We used covariates-adjusted general estimating equations for the analyses.

**Results:**

Longitudinal results showed that participants with weakness increased the likelihood of outpatient visits (odds ratio (OR) = 1.13; 95% confidence interval (CI) = 1.01–1.27), inpatient visits (OR = 1.51; 95% CI = 1.32–1.73), and unmet hospitalisation needs (OR = 1.44; 95% CI = 1.19–1.79) than their counterparts. Participants with weakness increased the number of outpatient visits (incidence rate ratio (IRR) = 1.29; 95% CI = 1.11–1.51) and the number of inpatient visits (IRR = 1.39; 95% CI = 1.10–1.61). Participants with HGS asymmetry increased the likelihood of unmet hospitalisation needs (OR = 1.19; 95% CI = 1.03–1.43) than their counterparts. The results of the impact of every one-kilogramme (kg) increase in HGS on health care utilisation indicated consistent results. The associations were similarly observed irrespective of gender.

**Conclusions:**

Chinese older adults with weakness or HGS asymmetry used more health care. Interventions for improving muscle strength and correcting strength asymmetry are highly recommended, with the potential to considerably save households and health care systems.

The increase in life expectancy and the decline in the fertility rate have made ageing an inevitable trend worldwide [1]. In line with this, China has also stepped into the ageing society since 2000, and the ageing process is much faster than that in other low- and middle-income countries [2]. According to the National Bureau of Statistics of China data, 264.02 million people were 60 years or older, accounting for 18.7% of the total population by 2020 [3]. Ageing society, coupled with the increasing demand for health care services, has become an important challenge for health care professionals and public health authorities in China [4]. Thus, improving our understanding of health care utilisation determinants may help develop effective strategies to reduce health care needs.

Handgrip strength (HGS) is largely based on neuromuscular function and is a reliable measure of neuromuscular integrity and muscle function [5]. Weakness (reduced muscle strength/low HGS) is associated with a variety of poor health outcomes, including chronic morbidities, functional disabilities, falls, fractures, cognitive decline, and all-cause mortality [6–8]. Moreover, HGS asymmetry, which is characterised by wide differences in HGS between hands, has also been shown to be associated with several adverse health outcomes, such as depression, anxiety, and cognitive impairment [9–11]. HGS indicates overall muscle strength and is associated with various clinically relevant health outcomes in older adults. Meanwhile, individuals’ health status is also associated with hospitalisation and health care utilisation [12]. Logically, HGS may predict health care utilisation in older adults. Previous studies have shown a significant relationship between HGS and health care costs [13–15]. For example, a cost-of-illness study in the Czech Republic, comprising 689 participants aged ≥70 years, suggested that low HGS was associated with an increased yearly health care cost of EUR 564 per person [16]. However, in China, previous studies mainly focus on how HGS impacts an individual’s health (i.e. chronic condition, mental health, and all-cause mortality) [17,18]; evidence on whether HGS could predict increased health care utilisation among Chinese older adults is limited. Understanding whether HGS is a significant predictor for increased health care utilisation among older adults could have important implications for screening and medical resource planning and policy.

To contribute to closing the existing research gaps, we aimed to examine the prospective association of HGS with health care utilisation among Chinese older adults (≥60 years) and to test whether these associations varied by gender. We hypothesised that weak individuals or individuals with HGS asymmetry would be more likely to use health care services and have higher unmet health care needs than their counterparts. Previous studies reported that there is a gender differential associated with HGS [19,20]. Hence, we expected that the impact of HGS on health care utilisation varied by gender.

## METHODS

### Study design and population

This study was embedded in the China Health and Retirement Longitudinal Study (CHARLS), a nationally respective longitudinal survey of Chinese people aged ≥45 years conducted in 28 provinces nationwide. The exact methodology of the CHARLS has been previously described [21]. Briefly, 17 708 Chinese middle-aged and older adults were enrolled in the baseline survey in 2011. Respondents were followed every two years, and a small set of new participants was included. Three follow-up surveys have been conducted so far: in 2013 (wave two; n = 18 605), 2015 (wave three; n = 21 095), and 2018 (wave four; n = 19 816). All participants or their legal representatives signed written informed consent forms to participate in the baseline and follow-up surveys. Informed consent was obtained from study participants before completing the study questionnaire. The study was approved by the Biomedical Ethics Committee of Peking University (IRB00001052-11015). To protect the confidentiality of personally identifiable information, all sensitive information is deleted, including specific addresses of individuals surveyed, telephone numbers, identification numbers, etc.

This study used data from baseline to wave four. We analysed data of baseline participants aged ≥60 who had at least one wave of HGS measured on both hands with information about hand dominance (right, left) and one or more follow-up waves of health care utilisation assessed in waves two to four (n = 6007).

### Measures

#### Handgrip strength

The HGS test protocol is detailed in the CHARLS Handgrip Strength Procedures Manual. In brief, handgrip strength was measured with a handheld dynamometer (Yuejian WL-1000, China) in kg. Those who have experienced surgery, inflammation, severe pain, or injury on one or both hands in the preceding six months were not asked to complete the HGS testing. The dominant hand of each participant was recorded. Participants were asked to stay in a standing position, hold the dynamometer at a right angle (90°), and squeeze the handle with their maximum effort for a few seconds. HGS was measured twice on each hand, alternating between hands, and the maximum reading from all four tests was used to reflect HGS.

Weakness was defined using the Asian Work Group for Sarcopenia 2019 consensus (maximal HGS<28 kg in male participants and <18 kg in female participants) [22]. The greatest HGS values from the dominant and nondominant hands were used to calculate the HGS ratio (nondominant HGS (kg)/dominant HGS (kg)). Although HGS varies between hands and is related to hand dominance, the ‘10% rule’ [23,24], which indicates that the HGS of the dominant hand is generally 10% greater than that of the non-dominant hand, was used to operationalise HGS asymmetry. Therefore, participants with an HGS ratio <0.9 or >1.1 were classified as having HGS asymmetry.

#### Health care utilisation

Health care utilisation outcomes include three aspects: 1) outpatient visits, 2) inpatient visits, and 3) unmet hospitalisation needs. Specifically, participants were asked the following questions: 1) ‘In the last month, have you visited a public hospital, private hospital, public health centre, clinical, or health worker’s or doctor’s practice, or been visited by a health worker or doctor for outpatient care?’, 2) ‘Have you received inpatient care in the past year?’. If they answered ‘yes’ to these screening questions, the participants were further asked their numbers of visits to outpatient and/or inpatient care. Unmet hospitalisation needs were measured by the question: ‘In the past year, did a doctor suggest that you needed inpatient care, but you did not get hospitalised?’

#### Covariates

The following sociodemographic and health-related characteristics were included in the analyses as potentially confounding variables based on the existing literature [9,10,25]: age (in years), sex (male, female), living residence (rural, urban), education achievement (primary school or below, middle school or above), marital status (married, other), household income per capita, health insurance (no insurance, new rural cooperative medical scheme, urban employee basic medical insurance, urban residents basic medical insurance, and other insurances), smoking (yes, no), alcohol use (yes, no), instrumental activities of daily living (IDAL), basic activities of daily living (BADL), and multimorbidity (number of conditions). Body mass index (BMI) was calculated by dividing the weight in kg by the height in square metres, then participants were categorised as underweight (BMI<18.5), normal weight (18.5≤BMI<23.9), overweight (24≤BMI<27.9), and obesity (BMI≥28) based on Chinese criteria. Sociodemographic and health-related indicators were included as covariates because these indicators impact muscle strength and are influential for our associations. Adjusting potential covariates in the model can reduce the error in the model and increase the power of the factor tests.

### Statistical analysis

Participants entered our study when HGS was first measured. Current health care utilisation and other covariates were assessed at each wave in which HGS was collected (i.e. every other wave). The outcome was health care utilisation at the next available wave. Time to follow-up between waves in which HGS was measured and the outcome was adjusted for in the analyses. The descriptive characteristics were presented as either frequency (%) or mean and standard deviation to characterise the study population. Independent *t* test and χ^2^ test were used to determine differences in the descriptive characteristics of weak and not weak participants and with and without HSG asymmetry participants for continuous and categorical variables, respectively.

We used separate generalised estimating equations (GEE) logistic regression models to examine the associations of 1) HGS weakness (reference: not weakness), 2) HGS asymmetry (reference: HGS symmetry), and 3) HGS per kg increase with future outpatient visits, future any inpatient visits, and future unmet hospitalisation needs. Similarly, separate GEEs poisson models were conducted to determine the associations of 1) HGS weakness (reference: not weakness), 2) HGS asymmetry (reference: HGS symmetry), and 3) HGS per kg increase with the future number of outpatient visits and the future number of inpatient visits. For the regression analyses, model one adjusts for only age and sex; model two adjusts for age, sex (when appropriate), residence, education, marital status, income, health insurance, smoking, alcohol use, IDAL, BADL, weight status, multimorbidity, and time between waves. For all GEEs, repeated measures were accounted for, and the outcomes for the next wave of participants were used. The effect estimates (odds ratio (OR) and incidence rate ratio (IRR)). The 95% confidence intervals (CIs) were reported, and *P*-value <0.05 was used to indicate statistical significance. We further conducted a subgroup analysis stratified by sex, using the same strategies as the aforementioned models. All analyses were conducted with SAS, version 9.4 (SAS Institute, Cary, North Carolina, USA). An α level of 0.05 was used for analyses.

## RESULTS

### Study population

The mean age of participants was 68.1 years (SD = 6.7), and 49.8% were male. Compared with participants without weakness, participants with weakness were older, had lower education levels, were not married, had lower household income, enrolled in no insurance or new rural cooperative medical scheme, had higher scores in IDAL and BADL, and had underweight or normal weight (*P* < 0.05). Additionally, compared with HGS symmetry, participants with HGS asymmetry were older, female, had higher scores in IDAL and BADL, and had underweight or normal weight (*P* < 0.05) (**Table 1**).

**Table 1:** Characteristics of study participants according to handgrip strength at baseline*

Characteristics	Overall (n = 6007)	HGS status		HGS status	
		**Not weak (n = 4889)**	**Weak (n = 1118)**	**Symmetry (n = 3158)**	**Asymmetry (n = 2362)**
Age in years, x̄ (SD)	68.1 (6.7)	67.2 (6.1)†	72.1 (7.6)†	67.8 (6.5)†	68.6 (7.0)†
Gender (male), n (%)	2989 (49.8)	2443 (50.0)	572 (51.2)	1646 (52.1)†	1124 (47.6)†
Residence (rural), n (%)	5223 (87.1)	4223 (86.5)‡	1000 (89.5)‡	2758 (87.4)	2041 (86.5)
Education level (primary school or below), n (%)	4945 (82.4)	3932 (80.4)†	1013 (90.9)†	2600 (82.4)	1942 (82.3)
Marital status (married), n (%)	4692 (87.1)	3947 (80.7)†	745 (66.6)†	2491 (78.9)	1823 (77.2)
Household income per capita, n (%)					
*Tertile 1 (lowest)*	1983 (33.3)	1530 (31.6)†	453 (40.7)†	1037 (33.1)	800 (34.2)
*Tertile 2 (middle)*	1983 (33.3)	1628 (33.7)†	355 (31.9)†	1032 (32.9)	783 (33.5)
*Tertile 3 (highest)*	1983 (33.3)	1679 (34.7)†	304 (27.3)†	1064 (34.0)	753 (32.2)
Health insurance, n (%)					
*None*	382 (6.4)	285 (5.9)†	97 (8.7)†	200 (6.3)	142 (6.1)
*New rural cooperative medical scheme*	4498 (75.2)	3622 (74.4)†	876 (79.0)†	2374 (75.3)	1766 (75.3)
*Urban employee basic medical insurance*	582 (9.7)	515 (10.6)†	67 (6.0)†	315 (10.0)	223 (9.5)
*Urban resident basic medical insurance*	246 (4.1)	204 (4.2)†	42 (3.8)†	121 (3.8)	100 (4.3)
*Other insurances*	270 (4.5)	243 (5.0)†	27 (2.4)†	142 (4.5)	113 (4.8)
Smoking (yes), n (%)	1826 (30.6)	1505 (31.0)	321 (29.2)	1007 (32.2)‡	682 (29.1)‡
Alcohol use (yes), n (%)	1810 (30.2)	1535 (31.4)†	275 (24.6)†	1038 (32.9)†	643 (27.3)†
IDAL, x̄ (SD)	1.2 (2.7)	1.0 (2.3)†	2.4 (3.8)†	1.0 (2.4)†	1.4 (2.8)†
BADL, x̄ (SD)	0.8 (1.9)	0.6 (1.6)†	1.5 (2.6)†	0.7 (1.6)‡	0.9 (1.9)‡
Weight status, n (%)					
*Underweight*	610 (10.5)	432 (9.1)†	178 (16.8)†	281 (9.2)‡	276 (12.1)‡
*Normal*	3150 (54.4)	2537 (53.6)†	613 (58.0)†	1710 (56.0)‡	1206 (52.9)‡
*Overweight*	1475 (25.5)	1288 (27.2)†	187 (17.7)†	766 (25.1)‡	586 (25.7)‡
*Obesity*	558 (9.3)	479 (10.1)†	79 (7.5)†	296 (9.7)‡	211 (9.3)‡
Multimorbidity, x̄ (SD)	1.6 (1.5)	1.6 (1.5)	1.7 (1.5)	1.6 (1.4)	1.7 (1.5)

BADL – basic activities of daily living, HGS – handgrip strength, IDAL – instrumental activities of daily living, SD – standard deviation, x̄ – mean

*Missing items: residence (n = 7), smoking (n = 47), alcohol use (n = 5), health insurance (n = 29), household income (n = 58), weight status (n = 214). *P*-values were calculated from an independent *t* test (continuous) and χ^2^ test (categorical variables).

†*P* < 0.001.

‡*P* < 0.05.

### Association between HGS status and health care utilisation

Results showed that participants with weakness increased the likelihood of outpatient visits (OR = 1.13; 95% CI = 1.01–1.27), inpatient visits (OR = 1.51; 95% CI = 1.32–1.73), and unmet hospitalisation needs (OR = 1.44; 95% CI = 1.19–1.79) than their counterparts. Participants with weakness increased the number of outpatient visits (IRR = 1.29; 95% CI = 1.11–1.51) and the number of inpatient visits (IRR = 1.39; 95% CI = 1.10–1.61) than their counterparts. Participants with HGS asymmetry increased the likelihood of unmet hospitalisation needs (OR = 1.19; 95% CI = 1.03–1.43) than those with HGS symmetry. We also examined the impact of every one kg increase in HGS on health care utilisation outcomes, which indicated consistent results (outpatient visits: OR = 0.98, 95% CI = 0.96–0.99; inpatient visits: OR = 0.96, 95% CI = 0.95–0.97; unmet hospitalisation needs: OR = 0.96, 95% CI = 0.95–0.97; the number of outpatient visits: IRR = 0.982; 95% CI = 0.98–0.99; the number of inpatient visits: IRR = 0.96, 95% CI = 0.95–0.98) (**Table 2**).

**Table 2:** Association between handgrip strength and health care utilisation*

Outcomes	HGS status		HGS status		HGS per kg increase
	**Not weak**	**Weak**	**Symmetry**	**Asymmetry**	
Any outpatient visit, OR (95% CI)					
*Model 1*	ref.	1.14 (1.01–1.28)†	ref.	1.01 (0.92–1.10)	0.993 (0.98–0.999)†
*Model 2*	ref.	1.13 (1.01–1.27)†	ref.	0.91 (0.90–1.09)	0.98 (0.96–0.99)†
Number of outpatient visits, IRR (95% CI)					
*Model 1*	ref.	1.31 (1.13–1.51)‡	ref.	1.01 (0.90–1.13)	0.988 (0.98–0.996)†
*Model 2*	ref.	1.29 (1.11–1.51)‡	ref.	0.99 (0.88–1.11)	0.982 (0.98–0.99)†
Any inpatient visit, OR (95% CI)					
*Model 1*	ref.	1.54 (1.35–1.75)‡	ref.	1.21 (0.88–1.29)	0.97 (0.96–0.98)‡
*Model 2*	ref.	1.51 (1.32–1.73)‡	ref.	1.03 (0.87–1.09)	0.96 (0.95–0.97)‡
Number of inpatient visits, IRR (95% CI)					
*Model 1*	ref.	1.47 (1.29–1.68)‡	ref.	1.02 (0.84–1.05)	0.97 (0.96–0.98)‡
*Model 2*	ref.	1.39 (1.10–1.61)‡	ref.	0.94 (0.84–1.05)	0.96 (0.95–0.98)‡
Unmet hospitalisation needs, OR (95% CI)					
*Model 1*	ref.	1.45 (1.19–1.76)‡	ref.	1.20 (1.02–1.41)†	0.96 (0.95–0.98)‡
*Model 2*	ref.	1.44 (1.19–1.79)‡	ref.	1.19 (1.03–1.43)†	0.96 (0.95–0.97)‡

CI – confidence interval, HGS – handgrip strength, IRR – incidence rate ratio, OR – odds ratio, ref – reference

*Model 1 was adjusted for age and sex. Model 2 was adjusted for age, sex, residence, education, marital status, income, health insurance, smoking, alcohol use, IDAL, BADL, weight status, multimorbidity, and time between waves.

†*P* < 0.05.

‡*P* < 0.001.

### Stratification analyses

Weakness was associated with outpatient visits, inpatient visits, and unmet hospitalisation needs in males and females. HGS asymmetry was associated with unmet hospitalisation needs only in females. Each one kg increase in HGS was associated with more future outpatient visits, inpatient visits, and unmet hospitalisation needs in males and females (**Table 3**).

**Table 3:** Association between handgrip strength and health care utilisation by sex*

Outcomes	HGS status		HGS status		HGS per kg increase
	**Not weak**	**Weak**	**Symmetry**	**Asymmetry**	
Male					
Any outpatient visit, OR (95% CI)					
*Model 1*	ref.	1.10 (1.02–1.30)‡	ref.	1.02 (0.89–1.16)	0.993 (0.98–0.998)†
*Model 2*	ref.	1.09 (1.01–1.29)‡	ref.	1.01 (0.88–1.15)	0.992 (0.98–0.998)†
Number of outpatient visits, IRR (95% CI)					
*Model 1*	ref.	1.40 (1.11–1.76)†	ref.	1.03 (0.87–1.21)	0.98 (0.97–0.99)†
*Model 2*	ref.	1.40 (1.09–1.80)†	ref.	1.01 (0.85–1.20)	0.98 (0.97–0.99)†
Any inpatient visit, OR (95% CI)					
*Model 1*	ref.	1.69 (1.41–2.01)‡	ref.	1.11 (0.83–1.12)	0.96 (0.95–0.98)‡
*Model 2*	ref.	1.62 (1.34–1.96)‡	ref.	1.05 (0.81–1.11)	0.96 (0.95–0.97)‡
Number of inpatient visits, IRR (95% CI)					
*Model 1*	ref.	1.63 (1.36–1.94)‡	ref.	1.29 (0.76–1.35)	0.97 (0.96–0.98)‡
*Model 2*	ref.	1.48 (1.23–1.78)‡	ref.	1.12 (0.75–1.23)	0.96 (0.95–0.98)‡
Unmet hospitalisation needs, OR (95% CI)					
*Model 1*	ref.	1.44 (1.08–1.91)†	ref.	1.00 (0.79–1.27)	0.97 (0.95–0.99)‡
*Model 2*	ref.	1.39 (1.12–1.87)†	ref.	1.04 (0.82–1.33)	0.97 (0.95–0.98)‡
Female					
Any outpatient visit, OR (95% CI)					
*Model 1*	ref.	1.16 (1.11–1.36)†	ref.	1.32 (0.88–1.62)	0.994 (0.98–0.997)†
*Model 2*	ref.	1.15 (1.09–1.36)†	ref.	1.21 (0.86–1.31)	0.994 (0.98–0.996)†
Number of outpatient visits, IRR (95% CI)					
*Model 1*	ref.	1.23 (1.02–1.48)‡	ref.	1.11 (0.85–1.16)	0.99 (0.94–0.997)‡
*Model 2*	ref.	1.20 (1.01–1.46)†	ref.	0.97 (0.83–1.13)	0.96 (0.93–0.97)†
Any inpatient visit, OR (95% CI)					
*Model 1*	ref.	1.39 (1.15–1.67)‡	ref.	1.09 (0.85–1.15)	0.97 (0.95–0.98)‡
*Model 2*	ref.	1.33 (1.06–1.62)‡	ref.	1.00 (0.83–1.13)	0.96 (0.95–0.98)‡
Number of inpatient visits, IRR (95% CI)					
*Model 1*	ref.	1.30 (1.07–1.58)†	ref.	1.13 (0.84–1.16)	0.97 (0.96–0.99)‡
*Model 2*	ref.	1.24 (1.10–1.65)†	ref.	1.12 (0.83–1.15)	0.97 (0.96–0.99)‡
Unmet hospitalisation needs, OR (95% CI)					
*Model 1*	ref.	1.44 (1.10–1.89)†	ref.	1.43 (1.13–1.79)†	0.96 (0.95–0.98)‡
*Model 2*	ref.	1.40 (1.05–1.86)†	ref.	1.40 (1.11–1.77)†	0.96 (0.94–0.97)‡

CI – confidence interval, HGS – handgrip strength, IRR – incidence rate ratio, OR – odds ratio, ref – reference

*Model 1 was adjusted for age. Model 2 was adjusted for age, residence, education, marital status, income, health insurance, smoking, alcohol use, IDAL, BADL, weight status, multimorbidity, and time between waves.

†*P* < 0.05.

‡*P* < 0.001.

## DISCUSSION

The purpose of this study was to investigate the prospective association of HGS with health care utilisation among older Chinese adults and to test whether these associations varied by gender. Using nationally representative data from the CHARLS, results suggested that older adults with weakness and HGS asymmetry experienced an increased risk for outpatient and inpatient use and unmet hospitalisation needs. The associations were independent of gender as well.

Previous literature on weakness and health care utilisation suggested that weakness is associated with increased hospitalisation costs and longer inpatient lengths of stay [26–28]. For example, a UK population-based estimate (aged 71–80 years) reported that weakness (low HGS) older adults were more likely to use informal care, inpatient secondary care and primary care, and higher total annual costs in health care than older adults without muscle weakness [29]. Hamasaki et al. [25] conducted a retrospective cohort study to investigate the association of handgrip strength with hospitalisation and mortality in Japanese patients with type two diabetes (mean age = 64 years). The results showed that handgrip strength was significantly associated with mortality and hospitalisation in men and with hospitalisation in women. However, these previous studies were mainly conducted in developed countries. Our findings based on the nationally representative data in China supported this conclusion, and consistent with our hypothesis, the results indicated that weakness is associated with future outpatient and inpatient visits and future unmet hospitalisation needs. The exact mechanisms underlying the associations of HGS weakness with subsequent health care utilisation are poorly understood. A possible explanation may be that older adults who are weak are often susceptible to adverse health outcomes, such as a higher risk of falls, chronic morbidities, cognitive impairment, and so on, which result in significant medical needs, high health care utilisation and costs [7,30]. Further study is needed to determine the mechanisms.

Consistent with our hypothesis, the results also revealed that HGS asymmetry is associated with future health care utilisation (i.e. unmet hospitalisation needs) among older adults. Although natural differences in muscle strength exist between hands, the presence of HGS asymmetry may be an indicator of neuromuscular system deficits or unilateral pathology [31,32], which signifies deteriorating health and requires more frequent use of health resources. When HGS was used as a continuous variable, our results showed that each one kg increase in HGS was associated with future health care utilisation, including outpatient and inpatient visits, unmet hospitalisation needs, and the number of outpatient and inpatient visits. Measuring HGS is a simple, reliable, valid, and inexpensive method of strength assessment for population screenings [33]. Health care providers should be encouraged to use measures of handgrip strength to identify patients at risk for muscle weakness and inform targeted interventions to preserve strength.

Additionally, our findings add to the literature on whether the association between HGS and health care utilisation varied by gender. In our study, weak older adults experienced an increased risk of outpatient and inpatient use, unmet hospitalisation needs, and the risk of health care utilisation in the same direction in males and females, suggesting a strong and stable impact from HGS on health care burden across different population groups. For HGS asymmetry, the association between HGS asymmetry and unmet hospitalisation needs was significant only in females. Previous studies only investigated sex differences in the association of HGS asymmetry with health outcomes [34,35]. For example, Liu et al. [19] used data from the English longitudinal study of ageing and reported that HGS asymmetry increased the risk of multimorbidity in women only. Another longitudinal study among Americans aged ≥50 years observed that HGS asymmetry posted a greater risk of premature mortality for women only [36]. To our knowledge, this is the first large-scale prospective study to evaluate the sex-specific association between HGS asymmetry and health care utilisation, and there is no prior study on this issue for reference. We recommended more studies on this topic in future longitudinal studies. Additionally, we suggested that HGS asymmetry should be evaluated in HGS test protocols, which could improve the prognostic utility of handgrip dynamometers and better operationalisation of muscle function, especially in women.

The results of this study suggest that interventions aimed at reducing health care utilisation among older adults should target early screening and intervention for potentially modifiable weakness/strength asymmetry. Additionally, we recommend that policymakers or health professionals pay more attention to weak older adults and consider strategies to improve their muscle function training.

A major strength of this study is a nationally representative sample and prospective study design, which helps to provide convincing support for the hypothesised relationship. Second, exploration of the longitudinal associations, using repeated measures of outcomes and predictors throughout follow-up, enables an insightful understanding of the direction of the relationship between HGS and health care utilisation. Proper identification of the comprehensive association would help guide interventions aimed at preserving muscle strength and reducing the need for health care. Nevertheless, some limitations should be considered. First, although several important confounding variables have been included in the models, unmeasured covariates might have a potential confounding effect on the associations, such as nutrient status [37]. However, the prospective association of HGS with health care utilisation found in the present study provides complimentary evidence to earlier cross-sectional research. Second, participants who could not perform the HGS evaluation were excluded from this study. Therefore, our conclusion might not be generalised to the general older adults. Third, HGS may naturally vary between hands. A standardised HGS asymmetry cutoff value could be warranted.

## CONCLUSIONS

This study demonstrated that Chinese older adults with weakness or HGS asymmetry not only had more outpatient and inpatient visits but also had higher unmet hospitalisation needs than their counterparts. Future prospective studies should continue examining the impact of HGS on health care utilisation with varied populations and consider more confounders to confirm the current results. Interventions for improving muscle strength and correcting strength asymmetry are highly recommended, with the potential for considerable savings for households and health care systems.
